# The impact of enteric coating of aspirin on aspirin responsiveness in patients with suspected or newly diagnosed ischemic stroke: prospective cohort study: results from the (ECASIS) study

**DOI:** 10.1007/s00228-022-03391-2

**Published:** 2022-09-19

**Authors:** Mohamed Nabil Elshafei, Yahia Imam, Arwa Ebrahim Alsaud, Prem Chandra, Aijaz Parray, Mohamed S. Abdelmoneim, Khaldun Obeidat, Razan Saeid, Mohammad Ali, Raheem Ayadathil, Mouhand F. H. Mohamed, Ibtihal M. Abdallah, Shaban Mohammed, Naveed Akhtar, Mohammed Ibn-Masoud Danjuma

**Affiliations:** 1 Clinical Pharmacy Department, Hamad General Hospital, Hamad Medical Corporation, P.O. 3050, Doha, Qatar; 2 Neurology Department, Hamad General Hospital, Hamad Medical Corporation, Doha, Qatar; 3 Weill Cornell Medicine-Qatar, Doha, Qatar; 4 Internal Medicine Department, Hamad General Hospital, Hamad Medical Corporation, Doha, Qatar; 5 Biostatstics Section, Medical Research Center, Hamad Medical Corporation, Doha, Qatar; 6 The Neuroscience Institute, Academic Health Systems, Hamad Medical Corporation, Doha, Qatar; 7 Department of Pharmacy, Hamad Medical Corporation, Doha, Qatar; 8 College of Medicine, Qatar University, Doha, Qatar

**Keywords:** Plain aspirin, Enteric-coated aspirin, Aspirin response, Thromboxane B2, Stroke

## Abstract

**Background and purpose:**

Uncertainty remains regarding the impact of enteric-coated aspirin (EC-ASA) on secondary prevention of ischemic stroke compared to plain aspirin (P-ASA). Hence, this study was designed to investigate the effect of EC formulation on ASA response via evaluating thromboxane B2 (TXB2) levels in patients with suspected or newly diagnosed stroke.

**Methods:**

A prospective cohort study on suspected or newly diagnosed ischemic stroke patients who are aspirin-naive was conducted. Patients were received either EC aspirin or plain aspirin for at least 3 days. The primary outcome was the proportion of aspirin non-responsiveness between two groups (level of residual serum TXB2 associated with elevated thrombotic risk (< 99.0% inhibition or TXB2 > 3.1 ng/ml) within 72 h after three daily aspirin doses, while secondary outcomes were the incidence of early gastrointestinal tract (GIT) bleeding with the various aspirin preparations. (Trial registration: Clinicaltrials.gov NCT04330872 registered on 02 April 2020).

**Results:**

Of 42 patients, ischemic strokes were confirmed in both P-ASA (81%) and EC-ASA (67%) arms. ASA non-responsiveness showed no significant difference between the two formulations (P-ASA vs. EC-ASA; 28.6% vs 23.8%; *P* = 0.726). Univariate and multivariate logistic regression analysis showed that patients treated with EC-ASA were more likely to have a lower rate of non-responders compared to P-ASA (unadjusted OR 0.78; 95% CI 0.20, 3.11); with the risk highest in type 2 diabetic patients with HBA1c > 6.5% (adjusted OR 6; 95% CI 1.02, 35.27; *P* = 0.047). No incidence of GIT bleeding observed throughout the study.

**Conclusion:**

A significant proportion of ASA non-responsiveness was recorded regardless of ASA formulation administered. The increased risk of ASA non-responsiveness in diabetic patients needs further exploration by larger prospective studies.

## Introduction

Stroke is the second cause of death and the third cause of disability globally. Therefore, it necessitates immediate intervention and secondary prevention management of risk factors [1, 2]. Safety and efficacy of aspirin (ASA) in secondary prevention of cardiovascular disease including ischemic stroke were demonstrated in some studies and meta-analysis [1, 3–10]. As a result, current guidelines define a role for ASA in the prevention of recurrent stroke or transient ischemic attack (TIA) in patients with stroke [2, 11, 12]. According to the American Stroke Association guidelines, early use of ASA in ischemic stroke, within the first 48 h of symptom onset reduces the long-term risk of death and disability caused by acute ischemic stroke [13–15]. However, ASA effectiveness is limited with a relative risk reduction of 20 to 25% for ischemic stroke in patients with a prior stroke or TIA [16]. Previous studies have reported some degree of ASA resistance or ASA unresponsiveness in 20 to 30% of patients [17, 18]. One of the major causes of ASA resistance is ASA’s inability to inhibit thromboxane A2 (TXA2) biosynthesis leads to ASA ineffectiveness [17].

Aspirin exerts its major antithrombotic effect by irreversibly inhibiting the cyclooxygenase (COX) enzyme in the platelets which inhibits TXA2 biosynthesis and consequently, reduces serum level thromboxane B 2 (TXB2) (the stable TXA2 metabolite) [19]. TXA2-dependent platelet aggregation is varied along with Variable ASA doses (75 to 325 mg/day) [9] and different ASA formulations [20] as well. Enteric-coated aspirin (EC-ASA) shows low bioavailability as it delays and reduces ASA absorption compared to plain aspirin (P-ASA). Although some studies showed that platelet aggregation reduction is associated with the decreased bioavailability of EC-ASA, the effect of enteric coating on ASA resistance is still conflicting [21, 22]. Additionally, it is debated whether or not EC-ASA showed preferable gastric protection compared to the plain formulation [23–25].

To the best of our knowledge, no enough clinical trials have been investigated the comparative effectiveness of both ASA formulations in vulnerable patients such as ischemic stroke patients. Uncertainty remains regarding the prognostic effect of EC-ASA formulation on ASA-resistant thromboxane biosynthesis and the risk of gastrointestinal bleeding compared to P-ASA. Therefore, this study was designed to evaluate the effectiveness of the EC formulation of ASA on TXB2 levels and the risk of GIT bleeding in suspected or newly diagnosed stroke patients.

## Methodology

### Study design

Consecutive inpatients aged 18 years or older, admitted to Hamad General Hospital with suspected or confirmed ischemic stroke were prescribed either enteric coated or plain aspirin from August 2019 to January 2020 and enrolled in a prospective cohort study [26]. Eligible patients were given either EC-ASA or P-ASA on day 1. Patients have prescribed a plain loading dose of ASA (dispersible 300 mg followed by ASA 75 mg tablets, Actavis UK Ltd) or EC-ASA loading dose (300 mg followed by100 mg, ^®^ Bayer, Germany) for 3 days. Irrespective of the design of the study, all patients enrolled in the study have received the usual standard of stroke care management, and as they were kept on other treatments consistent with HMC ischemic stroke guidelines.

### Study population

Adult patients (18 to 75 years of age) who were ASA-naïve and newly diagnosed or suspected to have ischemic stroke were recruited in the study. Eligible participants had no prior history of ischemic heart disease, chronic kidney disease, or peripheral vascular disease. Additionally, they were not on any medications of antiplatelets, prostaglandin-related medications (non-steroidal anti-inflammatory drugs, misoprostol, and other anti-secretory drugs), or received thrombolytics as a uniform aspirin loading dose was not given as per hospital policy. Patients using salicylate-containing supplements or enteral feeding tubes were also excluded. The pre-identified eligible participants were consented, screened, counseled, and enrolled in the study. The choice of the individual aspirin type was done at the discretion of the attending physician.

All procedures performed in this study were in accordance with the ethical standards of the medical research center (MRC) of Hamad Medical Corporation (MRC number: 01–18-156) and with the 1964 Helsinki declaration ethical standards.

### Serum TXB2 assessment

Patients have been requested to give blood samples (10 ml) for estimation of the TXB2 level at baseline and the end of the study on the third day. Blood samples were kept labeled with anonymous patient-specific identifiers and kept in study refrigerators until analyzed. TXB2 levels were estimated in platelet-poor plasma of patients with a commercially available enzyme-linked immunosorbent assay (ELISA) kit (R & D Systems, Cat. No. KGE011) according to the manufacturer’s protocol.

### Clinical assessments and outcomes

Patients were assessed for incidence of stroke according to the American Heart Association/American Stroke Association definition [27], whilst TIA was defined as “a brief episode of neurologic dysfunction caused by focal brain or retinal ischemia, with clinical symptoms typically lasting less than 1 h and without evidence of acute infarction” on computed tomography (CT) brain [28]. Stroke mimics (SM) was defined as patients who initially present with stroke symptoms that were refuted based on clinical and imaging examination by a qualified stroke specialist, or the presence of other convincing medical explanation of symptoms in the presence of normal neuroimaging or a functional diagnosis is made that met with DSMV definition [29].

Stroke severity was assessed by the National Institute of Health Stroke Scale (NIHSS) [30]. Ischemic strokes were classified according to the initial presentation using the Bamford classification [31] and etiologically categorized based on the Trial of Org 10,172 in Acute Stroke Treatment (TOAST) classification [13]. Clinical outcomes were assessed by the modified rankin scale (mRS) [32] at discharge. Additionally, diabetes mellitus (DM) was defined as per American Diabetes Association criteria [33]; dyslipidemia and hypertension were defined as per these respective guidelines [34–37].

The proportion of ASA non-responders at day 3 was assessed as a primary outcome by defining the level of residual serum TXB2 associated with elevated thrombotic risk (< 99.0% inhibition or TXB2 level > 3.1 ng/ml). Additionally, the incidence of major and minor gastrointestinal bleeding due to ASA therapy during hospitalization as defined by the International Society on Thrombosis and Haemostasis (ISTH) was recorded as a secondary outcome [38].

### Statistical analysis

Descriptive statistics were used to summarize and determine the sample characteristics and distribution of participants’ data. The normally distributed data and results were reported with mean and standard deviation (SD); the remaining results were reported with median and inter-quartile range (IQR). Categorical data were summarized using frequencies and proportions. Associations between two or more qualitative data variables were assessed using the Chi-square (*χ*^2^) test or Fisher exact test as appropriate. Quantitative data between the two independent groups (ASA responders and non-responders) were analyzed using unpaired *t* or Mann–Whitney *U* test as appropriate. Paired *t* or Wilcoxon signed ranked test was used to compare the TXB2 level measured at baseline and post-baseline within each group.

Univariate and multivariate logistic regression analyses (controlling and adjusted for potential predictors and confounders) were applied to determine and assess the associations of potential risk factors and predictors (such as ASA types, diagnosis, age, gender, ethnicities, BMI, HbA1C levels, HDL, LDL, and other clinical features) with outcome variable ASA non-responders. The results of logistic regression analyses were presented as odds ratios (OR) with corresponding 95% confidence intervals (CI). A receiver operating characteristic (ROC) curve was calculated using significant predictors (as determined via multivariate regression) to assess model discrimination and predictive accuracy. ROC curves provide a comprehensive and visually attractive way to summarize the accuracy of predictions. All *P* values presented were two-tailed, and *P* values < 0.05 were considered as statistically significant. All statistical analyses were done using statistical packages SPSS 25.0 (SPSS Inc. Chicago, IL) and Epi-info (Centres for Disease Control and Prevention, Atlanta, GA) software.

## Results

Forty-two patients were recruited, 21 in the P-ASA arm, and 21 in the EC-ASA arm. The cohort was male predominated with 39/42 (93%) males. The mean age of the cohort was 51.5 ± 10.4 years (range 30–74 years.). The cohort was multi-ethnic with South Asians the predominant population (71.4%) followed by patients from the Middle East and North Africa (MENA)region (21.5%) and other ethnicities 7.1%.

Ischemic stroke (IS) was the predominant diagnosis in 72.8%, TIA was 2.4%, and stroke mimics (SM) were 23.8%. Deficits were minor with a mean NIHSS of 2.95 ± 2.84 (0–13). Vascular risk factors were prevalent with 71.4%, 64.3%, and 52.4% having DM, HTN, and dyslipidemia, respectively.

Most patients had no to mild disability with 71.2% having a mRS of 2 or less at discharge, (the mean mRS at discharge was 1.2 ± 1.6, range 0–4, median 0). The 2 arms were almost comparable (*P* > 0.05) apart from an increase in lacunar type strokes in the P-ASA arm as depicted by the Bamford classification.

Baseline characteristics of the P-ASA and EC-ASA cohorts are shown in Table 1. The mean TXB2 level at baseline was 16.42 ± 9.34 ng/ml (range 4.2–41.2) and a median of 14.34 ng/ml. The mean TXB2 level after ASA administration was 2.44 ± 1.22 ng/ml (range 4.2–41.2) and a median of 14.34 ng/ml. The mean difference between baseline TXB2 levels and post-exposure levels was 13.98 ± 8.68 amounting to a mean 82.46 ± 11.67% decrease (median 85.27%). A comparison between P-ASA and EC-ASA are shown in Table 2. There was no minor or major bleeding with either formulation over the short study period.

**Table 1:** Baseline characteristics of P-ASA and EC-ASA cohorts

	**P-ASA** ***n* = 21**	**EC-ASA** ***n* = 21**	***P* value**
Mean age (year)	52.2 ± 11.4 (median 54, IQR 43.5, 61)	50.8 ± 9.7 (median 52, IQR 44.5, 57)	0.663
BMI	26.21 + 3.51 (median 26.6, IQR 24.8, 28.2)	26.19 + 3.66 (median 27.3, IQR 23.6, 28.5)	0.992
Sex			
Male	21(100%)	18(85.7%)	0.231
Female	0(0%)	3(14.3%)	
Ethnicity			0.787
South Asian	14 (66.7%)	16 (53.3%)	
MENA	6 (28.6%)	3 (14.3%)	
Others	1 (4.8%)	2 (9.5%)	
Diabetes	17 (81%)	13 (61.9%)	0.172
Hypertension	14 (66.7%)	13 (61.9%)	0.747
Dyslipidaemia	9 (42.9%)	13 (61.9	0.216
Active smoking	8 (38.1%)	7 (33.3%)	0.747
Concomitant drugs			
Beta blockers	1 (4.8%)	3(14.3%)	0.610
Statins	0 (0%)	1(4.8%)	0.989
ACE/ARB inhibitor	2 (9.5%)	2(9.5%)	0.999
Diagnosis			
IS	17(81%)	14(66.7%)	
TIA	0(0%)	1(4.8%)	0.892
SM	4(19%)	6(28.6%)	
Bamford class *n* = 31	*n* = 17	*n* = 14	
TACI	0 (0%)	1 (7.1%)	
PACI	7 (41.2%)	3 (21.4%)	0.118
LACI	9 (52.9%)	3 (21.4%)	
POCI	1 (5.9%)	7 (50%)	
IS TOAST class *n* = 31	*n* = 17	*n* = 14	
SVD	11 (64.7%)	8 (57.1%)	
LVD	1 (5.9)	2 (14.3%)	
Cardioembolic	1 (5.9%)	1 (7.1%)	
Others	1 (5.9%)	1 (7.1%)	0.947
unknown	3 (17.6%)	2 (14.3%)	
Mean NIHSS	3 ± 2.8 (median 2, IQR 1, 5)	2.9 ± 3 (median 3, IQR 0.5, 4)	0.899
Mean mRS at discharge	1.24 + 1.58 (median 0, IQR 0, 3)	1.24 + 1.64 (median 0, IQR 0, 2.5)	0.978

Chi-square Fisher exact test was used for 2 × 2 tables and for tables more than 2 × 2, Yates corrected Chi-square test was applied in case of small cell frequencies (50% or more cells have expected frequencies < 5), whereas quantitative outcome measures were compared by using *t* test or Mann–Whitney *U* test (for skewed data) as appropriate to compute respective statistical *P* value

*IQR* Inter-quartile range, *BMI* body mass index

**Table 2:** Baseline and post-intervention TXB2 levels, the percentage decreased, and percentage responders

	**P-ASA**	**EC-ASA**	**Mean difference (95% CI)**	***P* value**
Mean baseline TXB2 (ng/ml)	16.03 + 10.25	16.81 + 8.57	− 0.78 (− 6.67, 5.11)	0.791
Mean post-ASA TXB2 (ng/ml)	2.65 + 1.21	2.22 + 1.22	0.43 (− 0.33, 1.19)	0.257
Difference (baseline-post baseline)	13.37 + 9.51	14.59 + 7.95	− 1.21 (− 6.67, 4.26)	0.657
Percentage decrease (%)	79.25 + 14.16	85.67 + 7.56	− 6.4 (− 13.50, 0.66)	0.074
Responders (TXB2 ≤ 3.1 ng/ml)	15 (71.4%)	16 (76.2%)	OR 0.78 (0.20, 3.11)	0.726

*CI* confidence interval, *OR* odds ratio

### Aspirin response

The mean percentage decrease in TXB2 was more with EC-ASA than with P-ASA (85.7% vs 79.3%); however, it did not reach statistical significance (*p* = 0.07) as shown in Fig. 2. Additionally, age, the severity of stroke on NIHSS did not statistically correlate with the decrease in TXB2 (*p* > 0.05). Eleven (26.2%, 95% CI 15.3%, 41.1%) of the entire cohort were ASA non-responders (TXB2 > 3.1 ng/ml in the second sample); this did not correlate with age, sex, presence of DM or HTN, the Bamford classification or the stroke etiology as depicted by the TOAST classification (*p* > 0.05). Furthermore, thromboxane B2 was not inhibited to equal or more than 99% in any patient in the study (as shown in Tables 4 and 5).

**Fig. 1:**
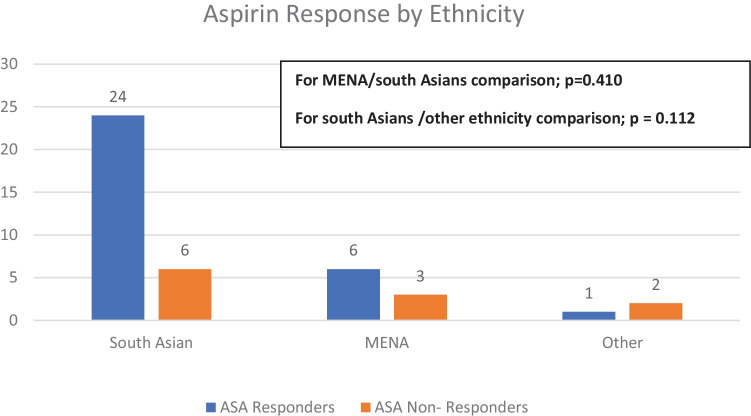
Percentage of ASA response on different race/ethnicity. ASA: aspirin, MENA: Middle East and North Africa

There was no statistically significance difference between non-responders in the P-ASA and EC-ASA groups (28.6% vs 23.8%) (*p* = 0.726). Interestingly, ethnicity correlated with a decrease in TXB2 with a higher percentage among non-South Asians as shown in Fig. 1. However, this was a modest correlation with ethnicity driving a 1.4% risk of decrease ASA response. Furthermore, the percentage of stroke or TIA patients labeled as non-responders where higher than stroke mimics appears to blunt the ASA response compared to non-strokes (stroke mimics) (32.3% vs 10%); however, the *p* value was statistically insignificant (*P* = 0.167).

The results of univariate and multivariate logistic regression analysis testing for each predictor and their possible association with ASA non-responders (< 99.0% inhibition or TXB2 level > 3.1 ng/ml) are presented in Table 3. Patients treated with EC-ASA were likely to have lower rates of non-responders compared to P-ASA (unadjusted OR 0.78; 95% CI 0.20, 3.11); however, this difference was statistically insignificant (*P* = 0.726). The risk of ASA non-response was found to be significantly higher in patients who had HbA1C > 6.5 compared to patients having HbA1C ≤ 6.5 (unadjusted OR 7.0; 95% CI 1.22, 40.1; *P* = 0.018). Compared to patients of younger age group (age ≤ 50 years), those who were in the age group more than 50 years had a twofold increased risk of ASA non-response (unadjusted OR 1.93; 95% CI 0.43, 8.69; *P* = 0.290). Similarly, patients with BMI > 25 (unadjusted OR 2.84; 95% CI 0.52, 15.46; *P* = 0.215) had a threefold increase risk. Compared with South Asians patients, MENA patients (unadjusted OR 2.0; 95% CI 0.39, 10.41; *P* = 0.410), and other ethnicities (unadjusted OR 8.0; 95% CI 0.62, 103.7; *P* = 0.112) were associated with an increased risk of ASA non-response. Both LDL > 3.5 mmol/L and HDL ≤ 1 mmol/L were found to have two- to fourfold increased risk associated with ASA non-response; however, these differences were statistically insignificant (*P* > 0.05). Patients presenting with ischemic stroke and large vessel disease etiology on TOAST classification both were likely to have an approximately fourfold increased risk associated with ASA non-response, though this did not reach statistical significance (*P* > 0.05). Similarly, other predictors and confounders such as female sex, smoking, hypertension, and platelet count were insignificantly (*P* > 0.05) associated with ASA non-responsiveness depicted in Table 3.

**Table 3:** Predictors and risk factors associated with aspirin non-responders: logistic regression analysis

**Variables/predictors**	**Aspirin non-responders *n*/*N* (%)**	**Unadjusted odds ratio (95% CI)**	***P* value**
**Aspirin type**			
Plain	6/21 (28.6%)	1.0 (Reference)	
EC	5/21 (23.8%)	0.78 (0.20, 3.11)	0.726
**Gender**			
Male	10/39 (25.6%)	1.0 (Reference)	
Female	1/3 (33.3%)	1.45 (0.12, 17.77)	0.770
**Age**			
≤ 50 years	3/16 (18.8%)	1.0 (Reference)	
> 50 years	8/26 (30.8%)	1.93 (0.43, 8.69)	0.390
**BMI**			
≤ 25	2/14 (14.3%)	1.0 (Reference)	
> 25	9/28 (32.1%)	2.84 (0.52, 15.46)	0.215
**Ethnicity**			
South Asians	6/30 (20%)	1.0 (Reference)	
MENA	3/9 (33.3%)	2.0 (0.39, 10.41)	0.410
Others	2/3 (66.7%)	8.0 (0.62, 103.7)	0.112
**Hypertension**			
No	4/15 (26.7%)	1.0 (Reference)	
Yes	7/27 (25.9%)	0.96 (0.23, 4.03)	0.958
**Platelets (10^9 per liter)**			
> 250	5/17 (29.4%)	1.0 (Reference)	
≤ 250	6/25 (24%)	0.76 (0.19, 3.04)	0.695
**HbA1C**			
≤ 6.5	2/22 (9.1%)	1.0 (Reference)	
> 6.5	7/17 (41.2%)	7.0 (1.22, 40.1)	0.018
**LDL (mmol/L)**			
≤ 3.5	6/29 (20.7%)	1.0 (Reference)	
> 3.5	3/11 (27.3%)	1.44 (0.29, 7.14)	0.656
**HDL (mmol/L)**			
> 1	2/19 (10.5%)	1.0 (Reference)	
≤ 1	7/21 (33.3%)	4.25 (0.76, 23.81)	0.085
**Toast criteria**			
SVD	7/19 (36.8%)	1.0 (Reference)	
LVD	2/3 (66.7%)	3.43 (0.26, 45.1)	0.398
Others	2/20 (10.0%)	0.19 (0.03, 1.08)	0.051
**Smoking**			
Non-smoker	5/22 (22.7%)	1.0 (Reference)	
Smoker	5/15 (33.3%)	1.7 (0.39, 7.36)	0.478
Ex-smoker	1/5 (20.0%)	0.85 (0.08, 9.44)	0.895
**Diagnosis**			
Mimics	1/10 (10.0%)	1.0 (Reference)	
Ischemic stroke	10/31 (32.3%)	4.29 (0.48, 38.64)	0.167

For some predictors, the sum is not equal to a total of 42 cases due to exclusion of some subcategories or missing observations

*CI* confidence interval, *OR* odds ratio, *BMI* body mass index, outcome variable: aspirin responders were considered as the reference group

Due to the smaller sample size, the width of 95% CI appears to be much wider that might limit the generalizability of these findings. The multivariable logistic regression analysis showed that only patients with HbA1C > 6.5 remained significantly associated with an increased (more than fivefold higher risk) risk of ASA non-response (adjusted OR 6.0; 95% CI 1.02, 35.27; *P* = 0.047) controlling and adjusting for all other potential confounder and predictors shown in Table 3. Finally, we computed a prediction model to evaluate the discriminative ability of potentially significant variables with statistical *P* < 0.10 on the occurrence of ASA non-response. Multivariate logistic regression indicated that the final model demonstrated a modest fit (area under the curve (AUC) = 0.722, 95% CI 0.53, 0.91) and included the potential predictors and risk factors as shown in Table 3.

## Discussion

To our knowledge, this study represents the first attempt at exploring the comparative efficacy and safety of EC-ASA vs P-ASA (as evidenced by the proportion of TXB2 inhibition) in a cohort of suspected acute stroke patients. We found about a quarter (26%) of the entire study cohort (regardless of ASA formulation) to be ASA non-responders (TXB2 > 3.1 ng/ml on the second sample) (Tables 4 and 5). Due to differences in the definition and methodology used to define ASA non-responsiveness, its reported prevalence from population estimates ranged from 5.5 to 60% [39]. In an Indian cohort of patients with myocardial infraction on dual antiplatelet agents, Pandey et al. reported about 18.4% rate of ASA non-responsiveness [40]. This appears consistent with the point estimates we have found in the south Asian cohort of our study (17.7%), but considerably less than the median for the entire cohort. This finding will suggest and support the earlier reported impact of ethnicity on the disposition of ASA in patients of South Asian extraction [40].

**Table 4:** Shows baseline and post-EC aspirin (3 doses) TXB2 levels and the percentage decreased

**Serial no.**	**Aspirin type**	**Sample A (TXB2 ng/ml)**	**Sample B (TXB2 ng/ml)**	**Difference**	**% decrease**
**1**	EC ASPIRIN	**18.93**	**3.07**	15.86	83.78
**2**	EC ASPIRIN	**23.73**	**1.78**	21.95	92.50
**3**	EC ASPIRIN	**21.49**	**3.35**	18.14	84.42
**4**	EC ASPIRIN	**36.80**	**3.97**	32.83	89.21
**5**	EC ASPIRIN	**12.57**	**3.95**	8.62	68.54
**6**	EC ASPIRIN	**17.40**	**2.79**	14.61	83.94
**7**	EC ASPIRIN	**7.76**	**0.46**	7.30	94.03
**8**	EC ASPIRIN	**12.48**	**2.65**	9.83	78.77
**9**	EC ASPIRIN	**7.96**	**0.72**	7.24	90.99
**10**	EC ASPIRIN	**10.89**	**1.63**	9.26	85.00
**11**	EC ASPIRIN	**8.06**	**0.65**	7.41	91.92
**12**	EC ASPIRIN	**20.96**	**2.15**	18.81	89.73
**13**	EC ASPIRIN	**10.53**	**2.21**	8.32	79.01
**14**	EC ASPIRIN	**17.64**	**4.42**	13.22	74.95
**15**	EC ASPIRIN	**23.51**	**0.63**	22.88	97.34
**16**	EC ASPIRIN	**4.19**	**1.18**	3.01	71.82
**17**	EC ASPIRIN	**8.37**	**0.63**	7.74	92.48
**18**	EC ASPIRIN	**23.57**	**2.24**	21.33	90.48
**19**	EC ASPIRIN	**34.19**	**3.55**	30.64	89.61
**20**	EC ASPIRIN	**17.49**	**2.221**	15.269	87.30
**21**	EC ASPIRIN	**14.48**	**2.42**	12.06	83.29
			**Average decrease (%)**		**85.67**

**Fig. 2:**
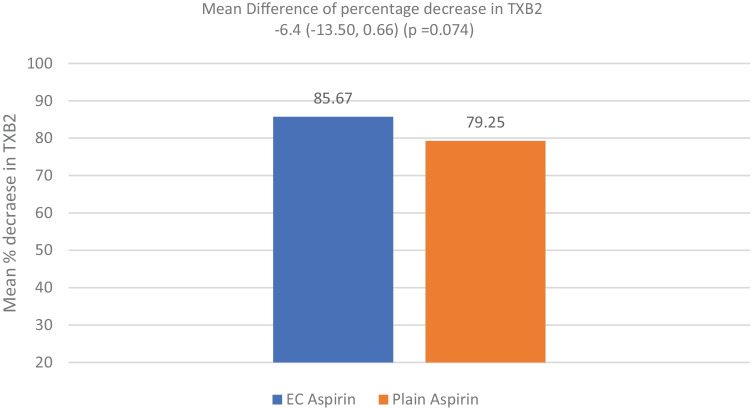
Mean percentage decrease in TXB2 of EC and plain aspirin

Adjustment for factors known to affect ASA pharmacokinetics (such as age, sex, diabetic morbidity status, body weight), HTN, thresholds of Bamford classification, primary stroke etiology as depicted by the TOAST classification (*p* > 0.05) resulted in no significant difference in the final point estimate with regards to TXB2 inhibition. However, in patients with DM, the level of glycemic control appears to impact the proportion of the response to ASA in patients with acute ischemic stroke. Despite HBA1c of 6.5 been an acceptable audit standard for good glycemic control, we found patients with HBA1c levels > 6.5 thresholds to be significantly at higher risk of ASA non-responsiveness compared to cohorts with levels < 6.5. Previous studies have already suggested reduced bioavailability of ASA leading to a state of “ASA resistance in type 2 diabetic patients [41]. How the level of glycemic control impacts this remains uncertain and needs to be explored by future prospective studies. Additionally, we found no significant difference in overall exposure to ASA between stroke patients on EC-ASA compared to those on P-ASA (as evidenced by the magnitude of TXB2 inhibition). Although a trend was apparent towards a decrease in TXB2 inhibition with EC-ASA compared to P-ASA (Tables 4 and 5) (Fig. 2), there was uncertainty regarding the final point estimate (85.7% vs 79.3%). Several recent studies from disparate patient populations have reported discordant outcomes following exposure to different ASA formulations (P-ASA or EC-ASA) [19, 21, 22, 42, 43]. Variability in these studies outcomes has been attributed to differences in patient populations, inconsistency in timing between ASA administration, and estimation of surrogate markers of ASA response amongst others. Bhatt et al. [22] is the most recent study to report on the evaluation of the risk of ASA non-responsiveness following exposure to EC-ASA.

**Table 5:** Shows baseline and post plain aspirin (3 doses) TXB2 levels and the percentage decreased

**Serial no.**	**Aspirin type**	**Sample A (TXB2 ng/ml)**	**Sample B (TXB2 ng/ml)**	**Difference**	**% decrease**
1	PLAIN ASPIRIN	**15.60**	**2.90**	12.70	81.43
2	PLAIN ASPIRIN	**29.09**	**3.64**	25.45	87.50
3	PLAIN ASPIRIN	**12.27**	**1.73**	10.54	85.87
4	PLAIN ASPIRIN	**4.33**	**2.44**	1.88	43.56
5	PLAIN ASPIRIN	**21.24**	**2.46**	18.79	88.44
6	PLAIN ASPIRIN	**14.19**	**1.22**	12.97	91.38
7	PLAIN ASPIRIN	**7.31**	**1.31**	6.00	82.10
8	PLAIN ASPIRIN	**9.21**	**1.33**	7.88	85.54
9	PLAIN ASPIRIN	**7.60**	**2.37**	5.23	68.82
10	PLAIN ASPIRIN	**18.05**	**2.02**	16.03	88.79
11	PLAIN ASPIRIN	**6.59**	**0.90**	5.70	86.40
12	PLAIN ASPIRIN	**12.84**	**2.07**	10.77	83.86
13	PLAIN ASPIRIN	**22.81**	**4.73**	18.09	79.29
14	PLAIN ASPIRIN	**18.04**	**2.31**	15.73	87.20
15	PLAIN ASPIRIN	**41.19**	**4.42**	36.77	89.26
16	PLAIN ASPIRIN	**7.32**	**1.97**	5.35	73.09
17	PLAIN ASPIRIN	**39.23**	**4.33**	34.90	88.95
18	PLAIN ASPIRIN	**6.76**	**4.24**	2.52	37.26
19	PLAIN ASPIRIN	**14.18**	**2.73**	11.45	80.78
20	PLAIN ASPIRIN	**8.99**	**1.95**	7.04	78.32
21	PLAIN ASPIRIN	19.81	4.673	15.14	76.41
			**Average decrease (%)**		**79.25**

In this study, EC-ASA was associated with about 52.8% non-responsiveness compared with P-ASA (15.8%). The difference in design between our study and that of Bhatt et al. [22] was rather interesting. Whilst our study cohort was comprised of exclusively suspected acute stroke patients, Bhatt et al.’s study cohorts were obese patients with type 2 DM [22]. It is noteworthy, that the ASA doses used by Bhatt et al. were uniform among different formulations of ASA (325 mg daily for 3 days) whereas our study allowed more pragmatic but different ASA maintenance doses. There is a reported difference in the absolute levels TXB2 [44] when the doses were doubled; however, this was not correlated with other surrogate markers of ASA responsiveness such as platelet aggregation or clinical effect [45]. This might in part explain better thromboxane inhibition associated with the enteric-coated formulation in contrast with Bhatt et al.’s study. However, further studies to ascertain the impact of various ASA doses on ASA responsiveness are needed. Furthermore, this study is different from Cox et al. (or indeed Frelinger or Mree et al.) [41, 43, 46] as it was conducted in patients with acute stroke or TIA rather than healthy volunteers or patients with stable CAD, as there is a considerable risk of stroke recurrence among admitted patients in initial period [47].

How differences in patients’ populations influence the outcomes of these two studies is not immediately clear. DM and obesity have variously been suggested as determinants of ASA non-responsiveness [48]. However, we found no significant difference in the rate of TXB2 inhibition stratified by DM or body weight. Probably, the acute setting of stroke as well as other socio-demographic factors (such as ethnicity) may have accounted for the differences in study outcomes. Bhatt et al. reported exclusively in a Caucasian cohort of patients, whilst our study population was comprised of a predominantly South Asian population. Body surface area has been well established as a PK determinant of drug response [49]. South Asians have a comparatively lower body surface than Caucasians, and this may have contributed to the significant proportion of non-responsiveness which was apparent following exposure to both formulations of ASA in our study. Additionally, it is likely the local and subsequent “spill-over” systemic inflammatory response seen in acute stroke patients may provide a milieu that could either potentially impede or augment ASA responsiveness [14]. In a study exploring the effect of serum levels of prothrombotic and or proinflammatory markers (such as CD40L, P-selectin, matrix metalloproteinase 9 [MMP-9]), interleukin (IL)-6, and intracellular adhesion molecule 1 (ICAM-1) on ASA and clopidogrel responsiveness (as evidenced by “point of care” platelet function assays) in ischemic stroke patients, Sternberg et al. reported that clopidogrel, in particular, was associated with both pro and anti-inflammatory effects; and that the “direct of inflammation” was a factor of the type of anti-platelet agent, and the lead time between antiplatelet administration and the timing of assay for surrogate markers of inflammation amongst others [50]. We additionally suspect inter-individual variability in the platelet recovery following ASA administration may have had an additional role in explaining our results, but our study design was not significantly powered to explore this.

Previous studies were constrained by uncertainty regarding the exact timing of ASA administration and the time of sampling of TXB2 levels [21, 43, 51, 52]. Differences in these studies design, choice of surrogate markers of ASA responsiveness, as well as the preference of platelet function methodology (PFA-100 device and the Ultegra-RPFA [RPFA]) with conventional light transmission aggregometry (LTA) have all contributed to the varying prevalence of apparent ASA non-responsiveness. Our study was not limited by this, as the timing of ASA administration and sampling for TXB2 levels was pre-specified in the study protocol. So, it is unlikely that this liability evident in previous studies had any impact on our study’s outcome.

Furthermore, even after adjustment for the severity of the stroke, we found no difference in the disposition of TXB2 levels between the two ASA formulations. However, having a stroke or TIA (compared to stroke-mimics) appears to paradoxically blunt response to ASA albeit with an uncertain final point estimate. The uncertainty of the exact point estimate we suspect may have to do with the relatively small sample size of our study population.

## Strength

The novelty of our report lies in its attempt at exploring the probable impact of enteric coating on the pharmacokinetic disposition of ASA in a cohort of suspected stroke patients. Despite its pilot design and lack of certainty regarding the point estimate between the two tested ASA formulations, it has raised some questions as were PK signals that would form the working hypothesis for future mechanistic as well as systematic studies.

## Limitations

Our study is limited by its small sample size which may have accounted for the uncertainty regarding the point estimates of TXB2 inhibition thresholds between the two ASA formulations. Simultaneously carrying out a platelet inhibition test would have provided a more robust context for interpretation of TXB2 inhibition levels and their impact on ASA non-responsiveness. Additionally, the use of different maintenance doses of ASA between the two arms while pragmatic and in line with international and local guidelines may be a potential confounder. Nevertheless, these are the limitations notwithstanding the outcome of this study.

## Conclusion

In a mixed population of acute stroke patients and stroke mimics, there was a significant proportion of ASA non-responsiveness regardless of ASA formulation administered (plain or enteric-coated). There was no difference in ASA effectiveness in terms of TXB2 inhibition between the two ASA formulations; however, the study was underpowered to detect non-inferiority. The increased risk of ASA non-responsiveness in diabetic patients HBA1c > 6.5 will need further exploration by larger prospective studies.

## Data Availability

Not applicable.
